# Supplementary material for: Quantum and non-local effects offer over 40 dB noise resilience advantage towards quantum lidar
Source: Nat Commun. 2022 Sep 26;13:5633. doi: 10.1038/s41467-022-33376-9 (PMC9512777; doi:10.1038/s41467-022-33376-9)
Supplement: Supplementary file 1 — Supplementary Information [file 41467_2022_33376_MOESM1_ESM.pdf]

Supplementary Information

Quantum and Non-local Effects Offer over 40dB Noise Resilience Advantage Towards Quantum Lidar

Phillip S. Blakey[1], Han Liu[1], Georgios Papangelakis[1], Yutian Zhang[1], Zacharie M. Léger[1], Meng Lon Iu[1], and Amr S. Helmy[1]

[1]Edward S. Rogers Faculty of Applied Science and Engineering, University of Toronto, Toronto, Ontario, Canada

Supplementary Note 1. Time-Frequency Entanglement and the Fractional Fourier Domain

Photon pairs generated through spontaneous parametric down conversion (SPDC) exhibit entanglement between there time and frequency degrees of freedom. For a perfectly monochromatic pump, due to the conservation of energy in the parametric process, the energy of each photon is uncertain however there sum is definite. Similarly, in the ideal case of perfect phase matching the emission of each photon is uncertain, however they are emitted simultaneously and so their time difference is definite. This is close analogy with the two mode squeezed vacuum state where the quadrature-phase of each mode is uncertain while their sum is zero and the in-phase quadrature of each mode is uncertain but their difference is zero. Specifically, one may draw a correspondance between $\hat{t}$ and $\hat{x}$ and $\hat{w}$ and $\hat{p}$. By using a pulsed pump, as we do in our experiment, there is some degree of uncertainty in the sum of the photon energies, which in turn results in an uncertainty in the difference in creation times. Thus, for our case a more apt analogy is with the finitely squeezed vacuum. Regardless, just as the temporal evolution of the quadratures rotates the basis in which we view the squeezing, the same may be done in the time-frequency domain [1]. From both the field quadrature perspective, as well as the time-frequency perspective, the basis rotation is best described by the Fractional Fourier Transform (FrFt). Importantly, in both cases the entanglement and thus correlations are invariant under the change of basis. While many methods exist to implement such a change of basis, in our experiment we use a normal dispersion fiber and an anomalous dispersion fiber applied to the signal and idler photons from a pulsed SPDC source.

Group velocity dispersion (GVD) is a phenomenon that closely relates to the time-frequency properties of light. An important application of GVD is frequency to time mapping using dispersive optics[2], which is usually understood as "different frequency components of light travels at different speeds in dispersive medium." However, it should be noted that this interpretation cannot be made exact: perfect mapping of frequency to time is impossible through dispersion since single frequency light has infinite temporal extent. Therefore, temporal detection after GVD must reflect both a fraction of temporal information and a fraction of spectral information of the un-dispersed pulse[3]. A useful tool to interpret and quantitatively analyze such compound information is the FrFT, which describes the continuous transition between the time domain amplitude to its Fourier transform. This formalism has been adopted for ultrafast pulse characterization[4].

The conversion between temporal correlation and frequency correlation is similar to two-mode squeezed vacuum light in which the signal and idler light have constant quadrature amplitude correlation when they experience opposite

phase shifts. However, unlike measuring quadrature or amplitude, the detection of time and frequency are fundamentally different in implementation and have different performance limiting factors. On one hand, the superconducting nanowire-based time-resolving single-photon detector has close to unity quantum efficiency and very low dark noise, but its temporal resolution $\simeq$ 10ps is too low compared to strong temporal correlation $\simeq$ 100fs entangled photons of entangled photon pairs. Time resolving detectors also have non-zero recovery time that results in jamming when the stream of photons are localized in time. On the other hand, frequency resolving single-photon detector based on dispersive diffraction and detector array [5] have relatively low optical efficiency detector efficiency, but they can handle higher photon flux due to their distributive detection nature. For correlation-based target detection, temporal resolution is needed to determine the target distance. However, since the strong temporal correlation of entangled photon pairs cannot be fully resolved with the detector, it is beneficial to convert some amount of immeasurable temporal correlation to measurable frequency correlation, to maximize correlation-based noise reduction.

In section* 1, we focus on analyzing the effects of GVD on a single photon based on the FrFt formalism. The single-photon regime is particularly interesting for two reasons: (1) in contrast to classical pulses that have arbitrary time and frequency distribution, single photons have well defined time and frequency operator[6], and (2) quantum entanglement of time and frequency can exist between a pair of single photons and such entanglement interacts with GVD in a nontrivial way. The FrFt modeling of single-photon GVD may also provide a useful theoretical tool for a class of single-photon quantum protocol, such as single-photon spectrography[7], dispersion-based quantum key distribution[8] and nonlocal dispersion cancellation[9]. The rest of this section* is organized as follows. We first apply the FrFt formalism to analyze the evolution of the time detection operator of a single photon. Then we use this result to generalize and characterize the property of time-frequency entanglement in photon pairs. We show that the correlation is preserved in the FrFt domain owing to entanglement being basis independent. We first show that dispersion can be modelled as a FrFT and that the single photon time operator evolves unitarliy according to

$$t' = U^{\dagger}\hat{t}U = \hat{t} + \beta^{(2)}L\hat{\omega} \tag{1}$$

where U is unitary operator describing group velocity dispersion with β^2 and L being the grop velocity dispersion and dispersion length.

We then show that for a bi-photon state

$$= \int dt_s dt_i f(t_s, t_i) a_s^{\dagger}(t_s) a_i^{\dagger}(t_i) |vac\rangle \tag{2}$$

where $f(t_s, t_i)$ is the (mod-square normalized) joint temporal amplitude,

$$f(t_s, t_i) = \sqrt{\frac{1}{2\pi\sigma_+\sigma_-} \exp(-\frac{t_s - t_i}{4\sigma_-^2} - \frac{t_s + t_i}{4\sigma_+^2})}. \tag{3}$$

the correlation in the FrFt domain is constant,

$$Corr(\hat{t}_s, \hat{t}_i) = Corr(\hat{t}'_s, \hat{t}'_i) = \frac{\sigma_+^2 - \sigma_-^2}{\sigma_+^2 + \sigma_-^2}. \tag{4}$$

Supplementary Note 2. Field and Operators

To avoid unnecessary complexity, we consider a single photon in a specific spatial-polarization mode. Then the observable and evolution of the photon can be expressed in terms of time and frequency domain annihilation operator, which are Fourier transform of each other:

$$a'(t) = \frac{1}{\sqrt{2\pi}} \int \tilde{a}'(\omega) \exp(-i\omega t) d\omega \qquad \tilde{a}'(\omega) = \frac{1}{\sqrt{2\pi}} \int a'(t) \exp(i\omega t) dt \tag{5}$$

For optical frequency, it is convenient to express both $a'(t)$ and $\tilde{a}'(\omega)$ in terms of center frequency shifted annihilation operators $a(t), \tilde{a}(\omega)$:

$$a'(t) = a(t)\exp(-i\omega_0 t) \qquad \tilde{a}'(\omega) = \tilde{a}(\omega - \omega_0) \tag{6}$$

The above Fourier transform relation still hold:

$$a(t) = \frac{1}{\sqrt{2\pi}}\int \tilde{a}(\omega)\exp(-i\omega t)d\omega \qquad \tilde{a}(\omega) = \frac{1}{\sqrt{2\pi}}\int a(t)\exp(i\omega t)dt \tag{7}$$

Single photon detection operator in time and frequency domain can be expressed as a weighted sum of photon density operator in time $a^\dagger(t)a(t)$ and frequency domain $\tilde{a}^\dagger(\omega)\tilde{a}(\omega)$ where $a^\dagger(t)$ and $\tilde{a}^\dagger(\omega)$ are the hermitian conjugates of $a(t)$ and $\tilde{a}(\omega)$ respectively :

$$\hat{t} = \int a^\dagger(t)a(t)tdt \qquad \hat{\omega} = \int \tilde{a}^\dagger(\omega)\tilde{a}(\omega)\omega d\omega \tag{8}$$

The effect of group velocity dispersion operator U can be modeled as a transform of frequency domain annihilation operator:

$$U^\dagger a(\omega)U = a(\omega)\exp(\frac{i\beta^{(2)}L\omega^2}{2}) \tag{9}$$

where $\beta^{(2)}$ and L are the group velocity dispersion and dispersion length, respectively.

Supplementary Note 3. Definition of Fractional Order Fourier Transform

As noted before, the FrFt proves to be a useful tool for analyzing the change of basis implemented through dispersion. We follow the definition of FrFt in [10]:

$$\mathcal{F}_{\alpha,T'}\{f(T')\}(T) = C(\alpha)\exp(\frac{i\pi T^2}{\tan\alpha})\int dT'\exp(\frac{i\pi T'^2}{\tan\alpha})\exp(-\frac{2i\pi TT'}{\sin\alpha})f(T') \tag{10}$$

$$C(\alpha) = \frac{\exp(-i\{\frac{\pi}{4}\text{sign}\{\sin\alpha\} - \frac{\alpha}{2}\})}{\sqrt{|\sin\alpha|}} \tag{11}$$

where T', T are dimensionless argument for the function before and after FrFt. The transform order α satisfy $0 < |\alpha| < \pi$. In what follows we shall use the following important properties of FrFt[10]:

$$\textbf{additivity:} \qquad \mathcal{F}_{\alpha_1,T'}\{\mathcal{F}_{\alpha_2,T''}\{f(T'')\}(T')\}(T) = \mathcal{F}_{\alpha_1+\alpha_2,T'}\{f(T')\}(T) \tag{12}$$

$$\textbf{multiplication rule:} \qquad \mathcal{F}_{\alpha,T'}\{\mathcal{F}_{-\alpha,T''}\{T''f(T'')\}(T')\}(T) = \cos\alpha f(T) - \frac{1}{i2\pi}\sin\alpha\frac{\partial}{\partial T}f(T) \tag{13}$$

$$\textbf{unitarity:} \qquad \int dT(\mathcal{F}_{\alpha,T'}\{f(T')\}(T))^*\mathcal{F}_{\alpha,T''}\{g(T'')\}(T) = \int dTf^*(T)g(T) \tag{14}$$

The conventional time-frequency Fourier transform in (7) can be written as a special case of FrFt. To do so one has to first normalized the dimension of time and frequency with some arbitrary time constant t_0:

$$T = t/t_0 \qquad A(T) = a(Tt_0) \qquad F = \frac{\omega t_0}{2\pi} \tag{15}$$

Then

$$\tilde{a}(\omega) = \frac{t_0}{\sqrt{2\pi}}\int dTA(T)\exp(2i\pi FT) = \frac{t_0}{\sqrt{2\pi}C(-\frac{\pi}{2})}\mathcal{F}_{-\pi/2,T}\{A(T)\}(F) \tag{16}$$

Supplementary Note 4. Modeling Dispersion using FrFt

To model the effect of dispersion on the time detection operator of a single photon, it suffices to consider the evolution of $a(t)$ under U:

$$U^\dagger a(t)U = \frac{1}{\sqrt{2\pi}}\int d\omega U^\dagger \tilde{a}(\omega)U\exp(-i\omega t) \tag{17}$$

$$= \frac{1}{\sqrt{2\pi}}\int d\omega \exp(\frac{i\beta^{(2)}L}{2}\omega^2)\tilde{a}(\omega)\exp(-i\omega t) \tag{18}$$

Use (16) and normalized variables in (15) (the two $1/\sqrt{2\pi}$ factors are cancelled by $d\omega/dF$) :

$$U^\dagger a(t)U = \frac{1}{\sqrt{2\pi}}\int d\omega \exp(\frac{2i\pi^2\beta^{(2)}L}{t_0^2}F^2)\tilde{a}(\omega)\exp(-i\omega t) \tag{19}$$

$$= \frac{1}{C(-\frac{\pi}{2})}\int dF \exp(\frac{2i\pi^2\beta^{(2)}L}{t_0^2}F^2)\exp(-2i\pi FT)\mathcal{F}_{-\pi/2,T'}\{A(T')\}(F) \tag{20}$$

Let:

$$\alpha = \text{arccot}(\frac{2\pi\beta^{(2)}L}{t_0^2}) \qquad \tau = T\sin\alpha \tag{21}$$

Then:

$$U^\dagger a(t)U = \int dF \exp(\frac{i\pi F^2}{\tan\alpha})\exp(-\frac{2i\pi F\tau}{\sin\alpha})\mathcal{F}_{-\pi/2,T'}\{A(T')\}(F) \tag{22}$$

$$= \frac{1}{C(\alpha)C(-\frac{\pi}{2})}\exp(\frac{-i\pi\tau^2}{\tan\alpha})\mathcal{F}_{\alpha,T'}\{\mathcal{F}_{-\pi/2,T''}\{A(T'')\}(T')\}(\tau) \tag{23}$$

$$= \frac{1}{C(\alpha)C(-\frac{\pi}{2})}\exp(\frac{-i\pi\tau^2}{\tan\alpha})\mathcal{F}_{\alpha-\frac{\pi}{2},T'}\{A(T')\}(\tau) \tag{24}$$

where we have used the additive property of FrFt. To simplify the notation, let$\gamma = \frac{\pi}{2} - \alpha$, then:

$$U^\dagger a(t)U = \frac{1}{C(\frac{\pi}{2}-\gamma)C(-\frac{\pi}{2})}\exp(\frac{-i\pi\tau^2}{\cot\gamma})\mathcal{F}_{-\gamma,T'}\{A(T')\}(\tau) \tag{25}$$

As can be seen, the effect of group velocity dispersion can be decomposed to first applying a FrFt to the input light, then apply temporal phase modulation $\exp(-\frac{2i\pi F\tau}{\sin\alpha})$. For temporal detection after dispersion, such phase modulation is not measurable.

Supplementary Note 5. Evolution of time detection operator

It is noted from (21) that the transform order γ depend on the normalization constant t_0. However, we will show now that temporal detection operator after dispersion relates to time and frequency operator of un-dispersed photon

in a way that is independent of t_0. The evolution of $\hat{t}$ can be expressed in terms of evolution of $a(t)$:

$$U^\dagger \hat{t} U \tag{26}$$

$$= \int U^\dagger a^\dagger(t) U U^\dagger a(t) U t dt \tag{27}$$

$$= |\frac{1}{C(\pi/2-\gamma)C(-\frac{\pi}{2})}|^2 \int dtt(\mathcal{F}_{-\gamma,T'}\{A(T')\}(\tau))^\dagger \mathcal{F}_{-\gamma,T''}\{A(T'')\}(\tau) \tag{28}$$

$$= \frac{t_0^2}{\cos\gamma^2}|\frac{1}{C(\pi/2-\gamma)C(-\frac{\pi}{2})}|^2 \int d\tau\tau\mathcal{F}_{\gamma,T'}\{A^\dagger(T')\}(\tau)\mathcal{F}_{-\gamma,T''}\{A(T'')\}(\tau) \tag{29}$$

where we used the property:

$$(\mathcal{F}_{\alpha,T'}\{A(T')\}(T))^\dagger = \mathcal{F}_{-\alpha,T'}\{A^\dagger(T')\}(T) \tag{30}$$

To simplify the above expression, we could try using the unitary property of FrFt. To do so, we need to convert operators $(A(T))$ to complex functions. We shall do this for the single photon case. Note that all pure single photon states can be expressed in the form of

$$|\phi\rangle = \int dt\phi(t)a^\dagger(t)|vac\rangle \tag{31}$$

with ϕ being mod-square normalized complex amplitude function. Then consider the quantity:

$$\text{temp} = \langle\psi| \int d\tau\tau\mathcal{F}_{\gamma,T'}\{A^\dagger(T')\}(\tau)\mathcal{F}_{-\gamma,T''}\{A(T'')\}(\tau)|\phi\rangle \tag{32}$$

$$= \int d\tau\tau\mathcal{F}_{\gamma,T'}\{\langle\psi| A^\dagger(T')\}(\tau)\mathcal{F}_{-\gamma,T''}\{A(T'')|\phi\rangle\}(\tau) \tag{33}$$

Since:

$$A(T)|\phi\rangle = a(Tt_0)\int dt\phi(t)a^\dagger(t)|vac\rangle = \phi(Tt_0)|vac\rangle \tag{34}$$

Then:

$$\text{temp} = \int d\tau\tau\mathcal{F}_{\gamma,T'}\{\psi^*(T't_0)\}(\tau)\mathcal{F}_{-\gamma,T''}\{\phi(T''t_0)\}(\tau) \tag{35}$$

$$= \int d\tau(\mathcal{F}_{-\gamma,T'}\{\psi(T't_0)\}(\tau))^*\tau\mathcal{F}_{-\gamma,T''}\{\phi(T''t_0)\}(\tau) \tag{36}$$

$$= \int d\tau\psi^*(\tau t_0)\mathcal{F}_{\gamma,T'}\{\tau\mathcal{F}_{-\gamma,T''}\{\phi(T''t_0)\}(T')\}(\tau) \tag{37}$$

where we used the unitary property of FrFt. Now we use the multiplication rule (13):

$$\text{temp} = \int d\tau\psi^*(\tau t_0)(\tau\cos(\gamma)\phi(\tau t_0) - \sin\gamma\frac{1}{i2\pi}\frac{\partial}{\partial\tau}\phi(\tau t_0)) \tag{38}$$

$$= \langle\psi| \int d\tau A^\dagger(\tau)(\tau\cos(\gamma)\phi(\tau t_0) - \sin\gamma\frac{1}{i2\pi}\frac{\partial}{\partial\tau}A(\tau))|\phi\rangle \tag{39}$$

Since ϕ, ψ are arbitrary, we have

$$U^\dagger\hat{t}U = \frac{t_0^2}{\cos\gamma^2}|\frac{1}{C(\pi/2-\gamma)C(-\frac{\pi}{2})}|^2 \int d\tau(\tau\cos(\gamma)A(\tau)^\dagger A(\tau) - \sin\gamma A(\tau)^\dagger\frac{1}{i2\pi}\frac{\partial}{\partial\tau}A(\tau)) \tag{40}$$

$$= \frac{t_0^2}{\cos\gamma}\int d\tau(\tau\cos(\gamma)A(\tau)^\dagger A(\tau) - \sin\gamma A(\tau)^\dagger\frac{1}{i2\pi}\frac{\partial}{\partial\tau}A(\tau)) \tag{41}$$

Consider the first term T_1 of the above expression:

$$T_1 := \frac{t_0^2}{\cos\gamma}\int d\tau\tau\cos(\gamma)A^\dagger(\tau)A(\tau) \tag{42}$$

$$= \cos^2\gamma\int dtta^\dagger(t\cos\gamma)a(t\cos\gamma) = \hat{t} \tag{43}$$

Now consider the second term T_2:

$$T_2 := \frac{i\tan(\gamma)t_0^2}{2\pi}\int d\tau A(\tau)^\dagger\frac{\partial}{\partial\tau}A(\tau) \tag{44}$$

$$= \frac{i\tan(\gamma)t_0^2}{2\pi}\int d(t\cos\gamma)a^\dagger(t\cos\gamma)\frac{\partial}{\partial t\cos\gamma}a(t\cos\gamma)) \tag{45}$$

$$= \beta^{(2)}L\int dta^\dagger(t)(i\frac{\partial}{\partial t})a(t) \tag{46}$$

Note that

$$\int d\tau a^\dagger(\tau)(i\frac{d}{d\tau})a(\tau) \tag{47}$$

$$= \frac{1}{2\pi}\int d\tau\int d\omega'\exp(i\omega'\tau)\tilde{a}^\dagger(\omega')(i\frac{d}{d\tau})\int d\omega''\exp(-i\omega''\tau)\tilde{a}(\omega'') \tag{48}$$

$$= \frac{1}{2\pi}\int d\tau\int d\omega'\exp(i\omega'\tau)\tilde{a}^\dagger(\omega')(i)(-i\omega'')\int d\omega''\exp(-i\omega''\tau)\tilde{a}(\omega'') \tag{49}$$

$$= \frac{1}{2\pi}\int d\tau\int d\omega'\int\omega''\exp(i(\omega'-\omega'')\tau)\tilde{a}^\dagger(\omega')\omega\exp(-i\omega''\tau)\tilde{a}(\omega'') \tag{50}$$

$$= \iint d\omega' d\omega''\tilde{a}^\dagger(\omega')\omega''\tilde{a}(\omega'')\delta(\omega'-\omega'') \tag{51}$$

$$= \hat{\omega} \tag{52}$$

Therefore, the final result is given by:

$$U^\dagger\hat{t}U = T_1 + T_2 = \hat{t} + \beta^{(2)}L\hat{\omega} \tag{53}$$

This is in agreement with the classical result [2].

Supplementary Note 6. Type I Phase-Matched SPDC Bi-Photon State

In order to model the effect of GVD on the photon pair it is necessary to derive the form of the joint-spectral amplitude of the two photons that characterises their correlation. In the lower pump power limit where higher order pair generation is negligible, and assuming a single spatial mode for the emitted photons, the Hamiltonian governing the Type I SPDC process can be written

$$\hat{H}_{PDC} = \nu\int_{-\infty}^{\infty} d\omega_s d\omega_i f(\omega_s,\omega_i)\hat{a}^\dagger(\omega_s)a^\dagger(\omega_i) + H.c. \tag{54}$$

where ν described the efficiency of the SPDC process and depends on the effective non-linearity and pump power, ω_s and ω_i are the signal and idler frequencies respectively, and $\hat{a}^\dagger(\omega_s)$ and $a^\dagger(\omega_i)$ are the corresponding bosonic mode

creation operators, and $f(\omega_s, \omega_i)$ is the joint-spectral amplitude (JSA) function characterizing the time-frequency structure of the bi-photon state [11]. The JSA is normalized such that $\int_{-\infty}^{\infty} d\omega_s d\omega_i |f(\omega_s, \omega_i)|^2 = 1$.
To first order the resulting state at the output facet of the waveguide neglecting the vacuum term and re-normalizing is given by

$$|\psi\rangle = \int d\omega_s d\omega_i f(\omega_s, \omega_i) \hat{a}^\dagger(\omega_s) a^\dagger(\omega_i) |vac\rangle \tag{55}$$

The form of the JSA arises from the requirement of conservation of energy, and conservation of momentum in the non-linear interaction and can be written as a product of the slowly varying pump envelope $\alpha(\omega_s + \omega_i)$ and phase matching function $\phi(\omega_s, \omega_i)$,

$$f(\omega_s, \omega_i) = \alpha(\omega_s + \omega_i)\phi(\omega_s, \omega_i). \tag{56}$$

We assume the pump pulse is a transform-limited Gaussian pulse so that,

$$\alpha(\omega_s + \omega_i) = \exp\left(-\frac{\tau_p^2}{2}(\omega_p^0 - \omega_s - \omega_i)^2\right), \tag{57}$$

where ω_p^0 is the pump center frequency and τ_p is the rms width of the pump envelope. The phase matching function is given by

$$\phi(\omega_s, \omega_i) = \int_0^L dz \chi(z) \exp\left(i\Delta k(\omega_s, \omega_i) z\right), \tag{58}$$

where $\Delta k(\omega_s, \omega_i) = k_p(\omega_s + \omega_i) - k_s(\omega_s) + k_i(\omega_i)$ is the phase-mismatch of the pump, signal, and idler waves, L is the length of the nonlinear medium, and $\chi(z)$ gives the sign of the spatially varying effective non-linearity in the periodically poled medium. The polling term adds an additional term to the phase-mismatch given by $k_{QPM} = \frac{2\pi}{\Lambda}$, where Λ is spatial period of the polling which allows for phase-matching of the center wavelengths. When the center wavelengths are phase-matched the phase matching function takes the form

$$\phi(\omega_s, \omega_i) = \frac{1}{L} sinc\left(\frac{\Delta k(\omega_s, \omega_i) L}{2}\right) e^{i\Delta k(\omega_s, \omega_i)\frac{L}{2}}. \tag{59}$$

Since the PPLN waveguide is degenerate Type I quasi-phase matched, the usual expansion of the phase mismatch to first order does not suffice and we must consider the group velocity dispersion terms. We expand the phase mismatch $\Delta k(\omega_s, \omega_i)$ to second order to get

$$\Delta k(\omega_s, \omega_i) = (\beta_{1,p} - \beta_1)(\omega_s + \omega_i - \omega_p) - \frac{1}{2}\beta_2\left[\left(\omega_s - \frac{\omega_p}{2}\right)^2 + \left(\omega_i - \frac{\omega_p}{2}\right)^2\right] + \frac{1}{2}\beta_{2,p}(\omega_s + \omega_i - \omega_p)^2, \tag{60}$$

where $\beta_n = \left.\frac{d^n k(\omega)}{d\omega^n}\right|_{\omega = \frac{\omega_p}{2}}$ and $\beta_{n,p} = \left.\frac{d^n k(\omega)}{d\omega^n}\right|_{\omega = \omega_p}$. We take the approximation that $\beta_{2,p} \approx 2\beta_2$ to get

$$\Delta k(\omega_s, \omega_i) = (\beta_{1,p} - \beta_1)(\Omega_s + \Omega_i) - \frac{\beta_2}{2}(\Omega_s - \Omega_i)^2$$

Where $\Omega_s = \omega_s - \frac{\omega_p^0}{2}$ and $\Omega_i = \omega_i - \frac{\omega_p^0}{2}$. Substitution into (56) and normalizing yields,

$$f(\Omega_s, \Omega_i) = \sqrt{\frac{4\sigma_+\sigma_-}{\pi}} \exp\left(-\sigma_+^2(\Omega_s + \Omega_i)^2 - \sigma_-^2(\Omega_s - \Omega_i)^2\right) \tag{61}$$

Where $\sigma_+^2 \approx \frac{1}{\tau_p^2+\frac{L^2}{10}(\beta_{1,p}-\beta_1)^2}$ and $\sigma_-^2 \approx \frac{12}{L\beta_2}$ [12]. It is useful to make the change of variables $\omega_s = \Omega_s + \frac{\omega_p}{2}$ and $\omega_i = \Omega_i + \frac{\omega_p}{2}$ to center the JSA at zero frequency and write the state

$$|\psi\rangle = \int d\omega_s d\omega_i f(\omega_s, \omega_i) \hat{a}^\dagger(\Omega_s)\hat{a}^\dagger(\Omega_i) \tag{62}$$

It is useful also to define the time-domain creation and annihilation operators

$$\hat{a}^\dagger(t) = \frac{1}{\sqrt{2\pi}} \int_{-\infty}^{\infty} d\omega e^{i\omega t} \hat{a}^\dagger(\omega). \tag{63}$$

The state of the SPDC photons at the output of the waveguide can then be re-written in the time domain as

$$|\psi\rangle = \int_{-\infty}^{\infty} dt_p dt_r f(t_p, t_r) e^{\frac{i}{2}\omega_p^{(0)}(t_p+t_r)} \hat{a}^\dagger(t_p)\hat{a}^\dagger(t_r,) \tag{64}$$

where $f(t_p, t_r) = \sqrt{\frac{1}{4\pi\sigma_+\sigma_-}} \exp\left(-\frac{(t_p+t_r)^2}{16\sigma_+^2} - \frac{(t_p-t_r)^2}{16\sigma_-^2}\right)$ is the joint-temporal amplitude function. This formalism gives us the tools to mathematically describe how dispersion affects the coincidence detection probabilities of the probe and reference photon in the pulsed pump regime.

Supplementary Note 7. GVD and Time-Frequency Entanglement

The state of a time-frequency entangled photon pair can then be written as the following:

$$|\text{pair}\rangle = \int dt_s dt_i f(t_s, t_i) a_s^\dagger(t_s) a_i^\dagger(t_i) |vac\rangle \tag{65}$$

where $f(t_s, t_i)$ is the (mod-square normalized) joint temporal amplitude. Equivalent expression can be obtained in the frequency domain, with the time domain operators replaced by frequency domain operators and $f(t_s, t_i)$ replaced by its Fourier transform. To simplify the analysis, we choose $f(t_s, t_i)$ to be a bivariate Gaussian function:

$$f(t_s, t_i) = \sqrt{\frac{1}{2\pi\sigma_+\sigma_-} \exp(-\frac{t_s - t_i}{4\sigma_-^2} - \frac{t_s + t_i}{4\sigma_+^2})} \tag{66}$$

Such a state is similar to two mode squeezed vacuum state in the quadrature-amplitude degree of freedom. The following correlation relation can calculated from the above expression:

$$\text{COV}(\hat{t}_s, \hat{t}_i) = \langle\text{pair}|\, \hat{t}_s \hat{t}_i \,|\text{pair}\rangle = \sigma_+^2 - \sigma_-^2 \qquad \text{COV}(\hat{\omega}_s, \hat{\omega}_i) = \langle\text{pair}|\, \hat{\omega}_s \hat{\omega}_i \,|\text{pair}\rangle = \frac{1}{2\sigma_+^2} - \frac{1}{2\sigma_-^2} \tag{67}$$

$$\text{COV}(\hat{t}_s, \hat{t}_s) = \text{COV}(\hat{t}_i, \hat{t}_i) = \sigma_+^2 + \sigma_-^2 \qquad \text{COV}(\hat{\omega}_s, \hat{\omega}_s) = \text{COV}(\hat{\omega}_i, \hat{\omega}_i) = \frac{1}{2\sigma_+^2} + \frac{1}{2\sigma_-^2} \tag{68}$$

$$\text{COV}(\hat{\omega}_s, \hat{t}_i) = \text{COV}(\hat{\omega}_i, \hat{t}_s) = 0 \tag{69}$$

One can quantify the correlation between time and frequency as correlation $Corr$:

$$Corr(\hat{t}_s, \hat{t}_i) = \frac{\text{COV}(\hat{t}_s, \hat{t}_i)}{\sqrt{\text{COV}(\hat{t}_s, \hat{t}_s)\text{COV}(\hat{t}_i, \hat{t}_i)}} = \frac{\sigma_+^2 - \sigma_-^2}{\sigma_+^2 + \sigma_-^2} \tag{70}$$

$$Corr(\hat{\omega}_s, \hat{\omega}_i) = \frac{\text{COV}(\hat{\omega}_s, \hat{\omega}_i)}{\sqrt{\text{COV}(\hat{\omega}_s, \hat{\omega}_s)\text{COV}(\hat{\omega}_i, \hat{\omega}_i)}} = \frac{-\sigma_+^2 + \sigma_-^2}{\sigma_+^2 + \sigma_-^2} \tag{71}$$

Despite the different dimension of time and frequency, the correlation in time and anti-correlation in frequency have equal magnitude. This is not a coincidence. To see this, consider time detection of dispersed signal photon and anti dispersed idler photon. Denote corresponding operators are $\hat{t}'_s$ and $\hat{t}'_i$ (note that $U^\dagger = U^{-1}$ represents negative dispersion):

$$\hat{t}'_s = U^\dagger \hat{t}_s U \qquad \hat{t}'_i = U \hat{t}_i U^\dagger \tag{72}$$

Then:

$$\mathrm{COV}(\hat{t}'_s, \hat{t}'_i) = \mathrm{COV}(\hat{t}_s + \hat{\omega}_s \beta^{(2)} L, \hat{t}_i - \hat{\omega}_i \beta^{(2)} L) \tag{73}$$

$$= \mathrm{COV}(\hat{t}_s, \hat{t}_i) - (\beta^{(2)} L)^2 \mathrm{COV}(\hat{\omega}_s, \hat{\omega}_i) = (\sigma_+^2 - \sigma_-^2)(1 + \frac{(\beta^{(2)} L)^2}{2\sigma_+^2 \sigma_-^2}) \tag{74}$$

$$\mathrm{COV}(\hat{t}'_s U, \hat{t}'_s) = \mathrm{COV}(\hat{t}'_i U, \hat{t}'_i) = (\sigma_+^2 + \sigma_-^2)(1 + \frac{(\beta^{(2)} L)^2}{2\sigma_+^2 \sigma_-^2}) \tag{75}$$

$$Corr(\hat{t}'_s, \hat{t}'_i) = \frac{\mathrm{COV}(\hat{t}'_s, \hat{t}'_i)}{\sqrt{\mathrm{COV}(\hat{t}'_s, \hat{t}'_s)\mathrm{COV}(\hat{t}'_i, \hat{t}'_i)}} = \frac{\sigma_+^2 - \sigma_-^2}{\sigma_+^2 + \sigma_-^2} \tag{76}$$

As can be seen, the time operator of dispersed signal and anti-dispersed idler have a constant amount of correlation, regardless of the magnitude of dispersion. Since dispersion is equivalent to FrFt for time domain detection, this result can be stated as signal and idler have constant correlation in the fractional order Fourier domain. This can also be intuitively understood as: one sacrifice some measurable temporal correlation to gain additional measurable frequency correlation. To show the reduction of temporal correlation, consider:

$$\langle \mathrm{pair} | (\hat{t}'_s - \hat{t}'_i)^2 | \mathrm{pair} \rangle = \mathrm{COV}((\hat{t}_s - \hat{t}_i) + \beta^{(2)} L(\hat{\omega}_s + \hat{\omega}_i), (\hat{t}_s - \hat{t}_i) + \beta^{(2)} L(\hat{\omega}_s + \hat{\omega}_i)) \tag{77}$$

$$= 4\sigma_-^2 + \frac{2(\beta^{(2)} L)^2}{\sigma_+^2} \tag{78}$$

As can be seen, the temporal uncertainty of $\hat{t}'_s - \hat{t}'_i$ increase with the amount of dispersion. But, because the total span of $\hat{t}'_s$ and $\hat{t}'_i$ increases as well, the total amount of correlation is unchanged.

Supplementary Note 8. CTD Mathematical Model

We compare both the DNCTD and NCTD to a classical analog to benchmark their performance. This classical analog consist of an intensity measurement at the same probe power as the DNCTD and NCTD schemes. The SNR is then defined as the ratio of the probe singles counts to noise single counts. To achieve the same probe power and noise power for the CTD scheme as the DNCTD and NCTD schemes we use the same measured data however instead of examining coincidences we look only at the singles counts. For this reason, we can write the probe singles counts in terms of the SPDC pair-rate ν which provides a useful metric for comparing with NCTD and DNCTD schemes. The SNR for the CTD scheme is then given by the probe photon rate divided by the noise photon rate or

$$SNR_{CTD} = \frac{\nu \tau_p}{N_b}, \tag{79}$$

where N_b is the number of recorded noise singles.

The question that of if this is indeed the optimal classical state to compare with under the same assumptions as the NCTD and DNCTD schemes is difficult to answer. The assumptions are: A completely phase-insensitive target-detection scheme; probe and noise power sufficiently small that at most one photon is expected within the

detector temporal resolution. Instead of addressing this problem directly we will motivate why this is a reasonable candidate. The requirement of phase-insensitivity requires that we perform an intensity measurement of the photons. However, we may correlate our photons with some classical signal. The most natural classical signal to correlate the photons with is the trigger signal from the pump laser. However, if we were to count coincidences between the probe/noise photons and the pump trigger the number of true coincidences would be $\nu\tau_p \cdot 1\text{trig/pulse}$ and the number of noise coincidences would be $N_b \cdot 1\text{trig/pulse}$ leaving the SNR unchanged. This is due to the fact that we have already assumed the noise is spectrally and temporally indistinguishable from the probe photon.

Supplementary Note 9. NCTD Mathematical Model

The NCTD model uses coincidence measurements to aid in distinguishing the probe from the coupled background noise. This differs from the case of coincidence counting with the pump trigger signal in the previous example as the number of coincidences between the probe and reference photons is dictated by the SPDC pair generation rate and the probe and reference channel transmissions and is given by $\nu\tau_p\tau_r$. Note, this assumes that the coincidence window is wider than the coincidence probability distribution width. That is, if a probe and reference photon make it through their corresponding channels, then their coincidence will be measured. The noise-reference coincidences are similarly given by $N_b\nu\tau_r$. This assumes perfect temporal overlap between the noise photons and the probe photons. The SNR is then given by

$$SNR_{NCTD} = \frac{\tau_p}{N_b}. \tag{80}$$

Supplementary Note 10. Noise Model

In order to model environmental noise in the probe arm of the target detection setup, we take the approximation that only one or zero noise photons can be present. That is the probability of a noise photon being measured is sufficiently small that the presence of two noise photons within the detector response time is negligible. Further, the noise photon is taken to have a spectral distribution identical to that of the probe photon. This second approximation is made to explicitly evaluate the enhancement dispersion compensation provides as out of band noise can, in theory, be filtered out. We take the broadband single photon creation operator to be of the form

$$\hat{A}^\dagger_{t_0} = \int d\omega h(t - t_0)\hat{a}^\dagger(t). \tag{81}$$

In practice, we take $t_0 = 0$ as the noise is made to be temporally indistinguishable from the probe photon. The requirement that the spectral distribution of the noise photon be identical to that of the probe photon can be written

$$\begin{aligned} h(\omega_i) &= \sqrt{\int_{\infty}^{\infty} |f(\omega_s, \omega_i)|^2 d\omega_s} \\ &= \sqrt{\frac{2\sqrt{2}\sigma_+\sigma_-}{\sqrt{\pi}\sqrt{\sigma_-^2 + \sigma_+^2}} \exp\left(-\frac{8\sigma_+^2\sigma_-^2}{\sigma_-^2 + \sigma_+^2}\omega_i^2\right)} \end{aligned} \tag{82}$$

The state of the noise photon is then given by

$$|\varphi\rangle_{noise} = \int d\omega h(\omega)\hat{a}^\dagger(\Omega)\,|vac\rangle \tag{83}$$

Now that a formalism for both the signal-idler photon pair and the noise source has been developed they must be combined to model possible noise photons in signal arm. Initially, before mixing the noise-signal state is just the

tensor product of the individual states $|\psi\rangle_{spdc} \otimes |\varphi\rangle_{noise}$. The method for combination of the noise and signal is shown in supplementary figure 1 below.

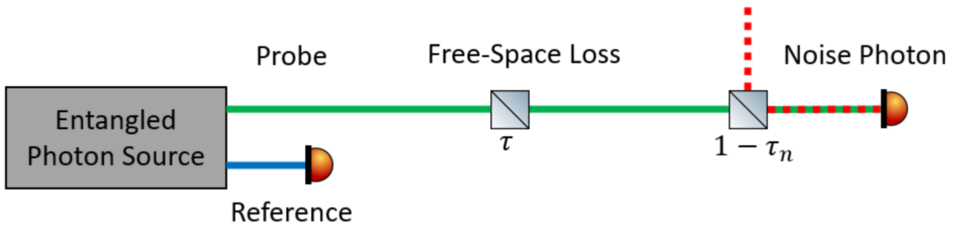

Supplementary Figure 1: Logical Layout for Noise/SPDC Model. The reference arm from the entangled photon source is assumed to be lossless. The signal arm experiences a free-space loss including target reflectively is modelled by a beam splitter of transmissivity τ. Noise addition is then modelled through another beam splitter of transmissivity $1-\tau_n$. The assumption made is that the signal photon either makes it to the detector–in which case no noise photon is present, or it is 'lost' and a noise photon replaces it.

First, free space loss is modeled as a beam splitter of transmisitivity τ acting on the signal creation operator. This beam spliter takes $\hat{a}_p^\dagger(\omega_s) \to \sqrt{\tau}\hat{a}_p^\dagger(\omega_s) + \sqrt{1-\tau}\hat{a}_{vac}^\dagger(\omega_s)$.

$$|\psi\rangle = \sqrt{\tau}\int d\omega_s d\omega_i d\omega h(\Omega) f(\omega_s,\omega_i)\hat{a}_p^\dagger(\Omega_s)\hat{a}_r^\dagger(\Omega_i)\hat{a}_n^\dagger(\Omega)|0\rangle + \sqrt{1-\tau}\int d\omega_s d\omega_i d\omega h(\Omega) f(\omega_s,\omega_i)\hat{a}_r^\dagger(\Omega_i)\hat{a}_n^\dagger(\Omega)\hat{a}_{vac}^\dagger(\Omega_s)|0\rangle \tag{84}$$

We now write this as a density operator and trace out by the unused port to get

$$\begin{aligned}\rho_{sys} = \tau\int d\omega_s d\omega_i d\omega_s' d\omega_i' f^*(\omega_s',\omega_i') f(\omega_s,\omega_i)\hat{a}_p(\Omega_s')\hat{a}_r(\Omega_i')|0\rangle\langle 0|\hat{a}_p^\dagger(\Omega_s')\hat{a}_r^\dagger(\Omega_i') \otimes \int d\omega' h^*(\omega')h(\omega)\hat{a}_n(\Omega')|0\rangle\langle 0|\hat{a}_n^\dagger(\Omega) \\ + (1-\tau)\int d\omega_s d\omega_i d\omega_i' f^*(\omega_s,\omega_i') f(\omega_s,\omega_i)\hat{a}_r(\Omega_i')|0\rangle\langle 0|\hat{a}_r^\dagger(\Omega_i) \otimes \int d\omega' h^*(\omega')h(\omega)\hat{a}_n(\Omega')|0\rangle\langle 0|\hat{a}_n^\dagger(\Omega)\end{aligned} \tag{85}$$

Next, the noise is combined with the signal by another beam spliter now with transmisitivity $1-\tau_n$. This corresponds to the Bogolibov transformations
$\hat{a}_p^\dagger(\Omega_s) \to (\sqrt{1-\tau}_n\hat{a}_p^\dagger(\Omega_s) + \sqrt{\tau_n}\hat{a}_{noise}^\dagger(\Omega_p))$ and $\hat{a}_{noise}^\dagger(\Omega) \to (-\sqrt{\tau_n}\hat{a}_p^\dagger(\Omega) + \sqrt{1-\tau_n}\hat{a}_{noise}^\dagger(\Omega))$ from which we define the new operators as $\hat{a}_1^\dagger(\Omega)$ and $\hat{a}_2^\dagger(\Omega)$ respectively. Using the inverse transforms we may write the product

$$\begin{aligned}\hat{a}_p^\dagger(\Omega_s)\hat{a}_n^\dagger(\Omega) &= [\sqrt{1-\tau_n}\hat{a}_1^\dagger(\Omega_s) - \sqrt{\tau_n}\hat{a}_2^\dagger(\Omega_s)][\sqrt{\tau_n}\hat{a}_1^\dagger(\Omega) + \sqrt{1-\tau_n}\hat{a}_2^\dagger(\Omega)] \\ &= \sqrt{\tau_n(1-\tau_n)}\hat{a}_1^\dagger(\Omega_s)\hat{a}_1^\dagger(\Omega) - \sqrt{\tau_n(1-\tau_n)}\hat{a}_2^\dagger(\Omega_s)\hat{a}_2^\dagger(\Omega) \\ &+ (1-\tau_n)\hat{a}_1^\dagger(\Omega_s)\hat{a}_2^\dagger(\Omega) - \tau_n\hat{a}_1^\dagger(\Omega)\hat{a}_2^\dagger(\Omega_s)\end{aligned} \tag{86}$$

We then trace out the unused beam splitter port corresponding to $\hat{a}_2^\dagger(\Omega)$. Next, we make the approximation to remain in the single photon regime. This means that there are only two possible situations; There is no noise photon and both of SPDC photons remain, and there is a noise photon that has 'replaced' the probe photon of the SPDC

pair. This is justified by the probabilities of a noise photon existing and an SPDC event occurring are individually small and the probability of both happening together is negligible. Specifically, this approximation corresponds to dropping the two-photon terms with $\sqrt{\tau\tau_n(1-\tau_n)}$ as a coefficient. Morover, we have assumed that all noise is added via this single beam splitter. This can be understood as a compounded beam splitter consisting of all noise sources along the propagation path. In this formalism, the incoming noise states would then be scaled accordingly to reflect there corresponding relative powers. We treat the noise as already ideally filtered and gated and so can simply consider a noise state that is indistinguishable from the probe photon. The resulting state after re-normalization is

$$\begin{aligned}\rho_{sys} =& \frac{\tau(1-\tau_n)^2}{\mathcal{A}} \int d\omega_i d\omega_i' d\omega_s d\omega_s' f^*(\omega_s', \omega_i') f(\omega_s, \omega_i) [\hat{a}_r^\dagger(\Omega_i)\hat{a}_1^\dagger(\Omega_s) |0,0\rangle \langle 0,0| \hat{a}_r(\Omega_i')\hat{a}_1(\Omega_s')] \\ &+ \frac{(\tau\tau_n^2 + (1-\tau)\tau_n)}{\mathcal{A}} \int d\omega d\omega' h^*(\omega') h(\omega) \hat{a}_1^\dagger(\Omega) |0\rangle \langle 0| \hat{a}_1(\Omega') \\ &\otimes \int d\omega_i d\omega_i' \Phi(\omega_i, \omega_i') \hat{a}_r^\dagger(\Omega_i) |0\rangle \langle 0| \hat{a}_r(\Omega_i') \end{aligned} \tag{87}$$

where $\mathcal{A} = 2\tau\tau_n^2 - 3\tau\tau_n + \tau + \tau_n$, and

$$\begin{aligned}\Phi(\omega_i, \omega_i') &= \int d\omega_p f(\omega_s, \omega_i) f^*(\omega_s, \omega_i') \\ &= \frac{2\sqrt{2}\sigma_+\sigma_-}{\sqrt{\pi}\sqrt{\sigma_+^2 + \sigma_-^2}} \exp\left(-\frac{\sigma_+^4(\omega_i - \omega_i')^2 + \sigma_-^4(\omega_i - \omega_i')^2 + 2\sigma_+^2\sigma_-^2(3\omega_i^2 + 2\omega_i\omega_i' + 3\omega_i'^2)}{2(\sigma_+^2 + \sigma_-^2)}\right)\end{aligned} \tag{88}$$

Supplementary Note 11. Dispersion Cancellation

We have already described how dispersion acts on the frequency annihilation operator. This may now be applied to the two components of the state separately. Letting the probe beam experience anomalous dispersion and the reference beam normal dispersion the noiseless term becomes

$$\begin{aligned}\rho_T^{disp} =& \frac{\tau(1-\tau_n)^2}{\mathcal{A}} \int d\omega_s d\omega_i d\omega_s' d\omega_i' f(\omega_s, \omega_i) \exp\left(-\frac{iL}{2}|\beta^{(2)}|\omega_s^2\right) \exp\left(\frac{iL}{2}|\beta^{(2)}|\omega_i^2\right) \hat{a}_p^\dagger(\Omega_s)\hat{a}_r^\dagger(\Omega_i) |0\rangle \\ &\langle 0| f^*(\omega_s', \omega_i') \exp\left(\frac{iL}{2}|\beta^{(2)}|\omega_s^2\right) \exp\left(-\frac{iL}{2}|\beta^{(2)}|\omega_i^2\right) \hat{a}_p(\Omega_s)\hat{a}_r(\Omega_i)\end{aligned} \tag{89}$$

In the time-domain this becomes

$$\rho_T^{disp} = \frac{\tau(1-\tau_n)^2}{\mathcal{A}} \int_{-\infty}^{\infty} dt_p dt_r dt_p' dt_r' f_{dispersed}(t_p, t_r) \hat{a}_p^\dagger(t_p)\hat{a}_r^\dagger(t_r) |0\rangle \langle 0| f_{dispersed}^*(t_p', t_r') \hat{a}_p(t_p')\hat{a}_r(t_r') \tag{90}$$

where, ignoring irrelevant phase terms,

$$\begin{aligned}f_{dispersed}(t_p, t_r) &= \mathcal{F}^{-1}\left[\exp\left(\frac{i}{2}\beta^{(2)}L\omega_s^2 - \frac{i}{2}\beta^{(2)}L\omega_i^2\right) f(\omega_s, \omega_i)\right] \\ &= \frac{\sqrt{4\sigma_+\sigma_-}}{\sqrt{\pi(16\sigma_+^2\sigma_-^2 + L^2(\beta^{(2)})^2)}} \exp\left(-\frac{2\sigma_+^2(t_p - t_r)^2 + 2\sigma_-^2(t_p + t_r)^2 + iL\beta^{(2)}(t_p^2 - t_r^2)}{32\sigma_+^2\sigma_-^2 + 2L^2(\beta^{(2)})^2}\right)\end{aligned} \tag{91}$$

We now examine the second term after the application of dispersion.

$$\rho_F^{disp} = \frac{(\tau\tau_n^2 + (1-\tau)\tau_n)}{\mathcal{A}} \int d\omega d\omega' h^*(\Omega')h(\Omega) \exp\left(-\frac{iL}{2}\beta^{(2)}\omega^2\right) \hat{a}_1^\dagger(\omega)\left|0\right\rangle\left\langle 0\right| \hat{a}_1(\omega') \exp\left(\frac{iL}{2}\beta^{(2)}\omega'^2\right)$$
$$\otimes \int d\omega_i d\omega_i' \Phi(\omega_i,\omega_i') \exp\left(\frac{iL}{2}\beta^{(2)}\omega_i^2\right) \hat{a}_r^\dagger(\Omega_i)\left|0\right\rangle\left\langle 0\right| \hat{a}_r(\Omega_i') \exp\left(-\frac{iL}{2}\beta^{(2)}\omega_i'^2\right) \tag{92}$$

Supplementary Note 12. Measurement and Coincidence Probability

The detection of the photons may be described through application of the POVMs corresponding to detection at detector P and R, given by

$$\hat{\Pi}_P(t_p) = \int dt g(t-t_p)\left|t\right\rangle_P \left\langle t\right|_P \qquad \hat{\Pi}_R(t_r) = \int dt g(t-t_r)\left|t\right\rangle_R \left\langle t\right|_R \tag{93}$$

The joint probability of a coincidence is given by is given by

$$P(t_1,t_2) = Tr_{R,P}\{\Pi_P(t_1)\Pi_R(t_2)\rho\} \tag{94}$$

Letting $g(t) = \frac{1}{\sqrt{2\pi}\sigma_{det}}\exp\left(-\frac{t^2}{2\sigma_{det}^2}\right)$ be the response function for the detector the probability of a true coincidence is then

$$\begin{aligned} P_T^{nd}(t_p,t_r) &= \frac{\tau(1-\tau_n)^2}{\mathcal{A}} \int dt_p' dt_r' |f(t_p,t_r)|^2 g(t_p-t_p')g(t_r-t_r') \\ &= \frac{\tau^2(1-\tau_n)^2}{\mathcal{A}} \frac{1}{2\pi\sqrt{(2\sigma_+^2+\sigma_{det}^2)(2\sigma_-^2+\sigma_{det}^2)}} \exp\left(-\frac{\sigma_+^2(t_p-t_r)^2+\sigma_-^2(t_p+t_r)^2+\sigma_{det}^2(t_p^2+t_r^2)}{2(2\sigma_+^2+\sigma_{det}^2)(2\sigma_-^2+\sigma_{det}^2)}\right). \end{aligned} \tag{95}$$

Similarly, the joint probability of a false coincidence is given by

$$\begin{aligned} P_F^{nd}(t_p,t_r) &= \frac{\tau\tau_n^2+(1-\tau)\tau_n^2}{\mathcal{A}} \frac{\sqrt{\sigma_+^2+\sigma_-^2}}{\sqrt{2\pi}\sqrt{4\sigma_+^2\sigma_-^2+\sigma_{dec}^2(\sigma_+^2+\sigma_-^2)}} \exp\left(-\frac{\sigma_+^2+\sigma_-^2}{2(4\sigma_+^2\sigma_-^2+\sigma_{dec}^2(\sigma_+^2+\sigma_-^2))}t_p^2\right) \\ &\times \frac{1}{\sqrt{2\pi}\sqrt{\sigma_{dec}^2+\sigma_+^2+\sigma_-^2}} \exp\left(-\frac{t_r^2}{2(\sigma_+^2+\sigma_-^2+\sigma_{dec}^2)}\right) \end{aligned} \tag{96}$$

For the dispersed case, the joint probability of a true coincidence is given by

$$\begin{aligned} P_T^d(t_p,t_r) &= \frac{\tau(1-\tau_n)^2}{\mathcal{A}} \int dt_p' dt_r' |f_{dispersed}(t_p',t_r')|^2 g(t_p-t_p')g(t_r-t_r') \\ &= \frac{\tau(1-\tau_n)^2}{\mathcal{A}} \frac{4\sigma_+\sigma_-}{\pi\sqrt{(16\sigma_+^2\sigma_-^2+8\sigma_-^2\sigma_{dec}^2+L^2(\beta^{(2)})^2)(8\sigma_+^2(2\sigma_-^2+\sigma_{dec}^2)+L^2(\beta^{(2)})^2)}} \\ &\exp\left(-\frac{2((16\sigma_+^4\sigma_-^2+l^2(\beta^{(2)})^2\sigma_+^2)(t_p-t_r)^2+(16\sigma_+^2\sigma_-^4+L^2(\beta^{(2)})^2\sigma_-^2)(t_p+t_r)^2+16\sigma_+^2\sigma_-^2\sigma_{dec}^2(t_p^2+t_r^2))}{(16\sigma_+^2\sigma_-^2+8\sigma_-^2\sigma_{dec}^2+L^2(\beta^{(2)})^2)(16\sigma_+^2\sigma_-^2+8\sigma_+^2\sigma_{dec}^2+L^2(\beta^{(2)})^2)}\right) \end{aligned} \tag{97}$$

and the joint probability of a false coincidence

$$
\begin{aligned}
P_F^d(t_p, t_r) = &\frac{\tau\tau_n^2 + (1-\tau)\tau_n^2}{\mathcal{A}} \frac{4\sigma_+\sigma_-\sqrt{\sigma_+^2 + \sigma_-^2}}{\sqrt{2\pi}\sqrt{(\beta^{(2)})^2 L^2(\sigma_+^2 + \sigma_-^2)^2 + 16\sigma_+^2\sigma_-^2(4\sigma_+^2\sigma_-^2 + (\sigma_{dec}^2)(\sigma_+^2 + \sigma_-^2))}} \\
&\times \frac{4\sigma_+\sigma_-}{\sqrt{2\pi}\sqrt{L^2(\beta^{(2)})^2(\sigma_+^2 + \sigma_-^2) + 16\sigma_+^2\sigma_-^2(\sigma_+^2 + \sigma_-^2 + \sigma_{dec}^2)}} \\
&\times \exp\left(-\frac{8\sigma_+^2\sigma_-^2(\sigma_+^2 + \sigma_-^2)t_p^2}{(\beta^{(2)})^2 L^2(\sigma_+^2 + \sigma_-^2)^2 + 16\sigma_+^2\sigma_-^2(4\sigma_+^2\sigma_-^2 + (\sigma_{dec}^2)(\sigma_+^2 + \sigma_-^2))}\right) \\
&\times \exp\left(-\frac{8\sigma_+^2\sigma_-^2 t_r^2}{16\sigma_+^2\sigma_-^2(\sigma_+^2 + \sigma_-^2 + \sigma_{dec}^2) + L^2(\beta^{(2)})^2(\sigma_+^2 + \sigma_-^2)}\right)
\end{aligned}
\tag{98}
$$

The joint coincidence probabilities provide an excellent way to visually depict how dispersion compensation effectively increases the detector temporal resolution and are plotted in supplementary figures 2a , 2b, 2c, 2d. The JSA bandwidths used are 121fs and 17.7ps FWHM estimated from the Ti:Sapphire laser and SHG spectrum of the PPLN waveguide, the dispersion parameter and length of the fibers are (18ps/nm · km, 5km), and detector time uncertainty is 50.0ps FWHM. The joint probability distribution for a true coincidence (supplementary figure 2a) is very weakly correlated–being predominantly dictated to the detector uncertainty. The joint probability distribution for a false coincidence (supplementary figure 2b) is perfectly uncorrelated and when the detector uncertainty is sufficiently large (as in our model), resembles very closely the probability distribution for a true coincidence. After the application of dispersion compensation to the probe and reference arms (supplementary figure 2c), the correlation between the probe and reference photons is recovered where dispersion occurs along the diagonal access and is mostly suppressed along the off-diagonal axis. In contrast, the joint probability of a false coincidence remains un-correlated after the applications of dispersion and experiences equal broadening along both axis. Since the coincidence probability distribution is the marginal distribution of the joint probability distribution along $t_p - t_r$, this has the effect of allowing noise that was previously indistinguishable from true coincidences to be filtered by choosing a coincidence window smaller than the width of the dispersed false coincidence peak.

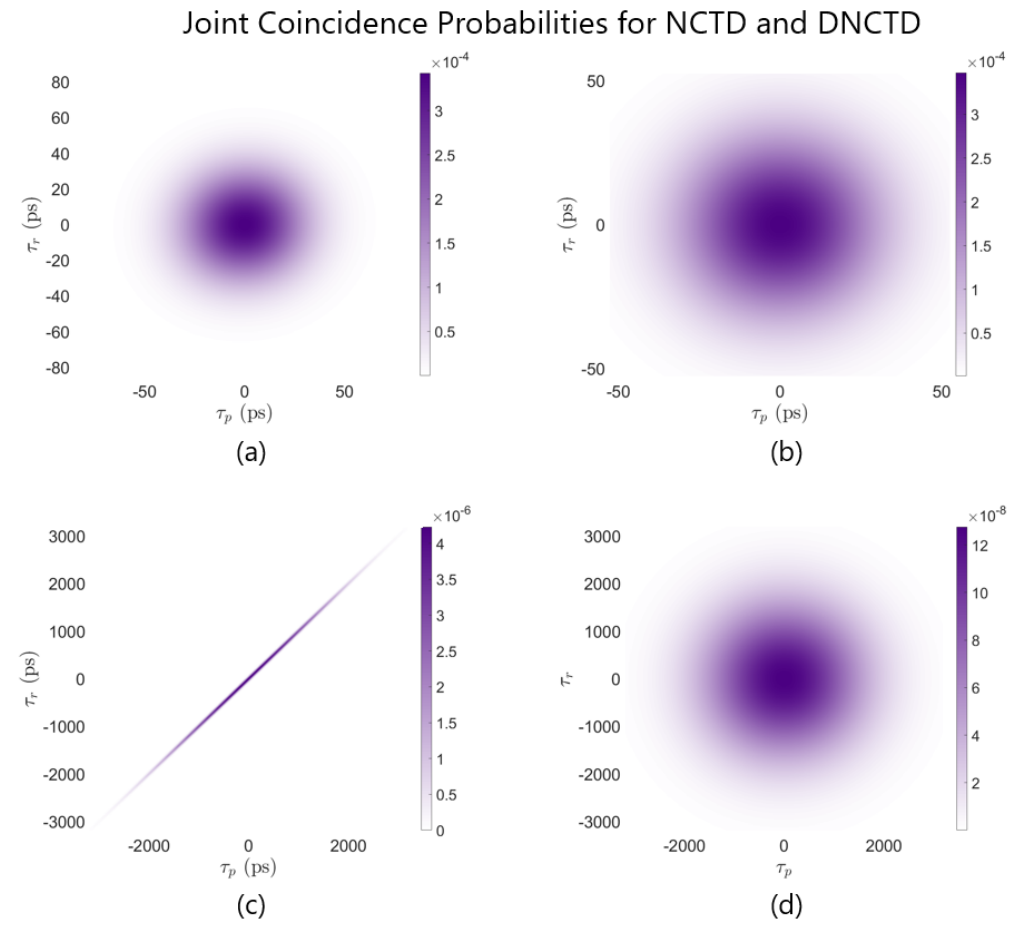

Supplementary Figure 2: Joint probability distributions for Coincidences Under Various Conditions (a) Joint probability distribution for a true coincidence $P_T^{nd}(t_p, t_r)$. (b) Joint probability distribution for a False coincidence $P_F^{nd}(t_p, t_r)$. (c) Joint probability distribution for a true coincidence with the application of dispersion $P_T^d(t_p, t_r)$. (d) Joint probability distribution for a false coincidence with the application of dispersion $P_F^d(t_p, t_r)$.

Supplementary Note 13. Coincidence Histograms

It is also very useful, both for understanding and experimental comparison, to calculate the coincidence histograms from the joint probability distributions. This can be done by finding the marginal distribution of the joint probability distributions along $t_p - t_r$ (supplementary figure 3).

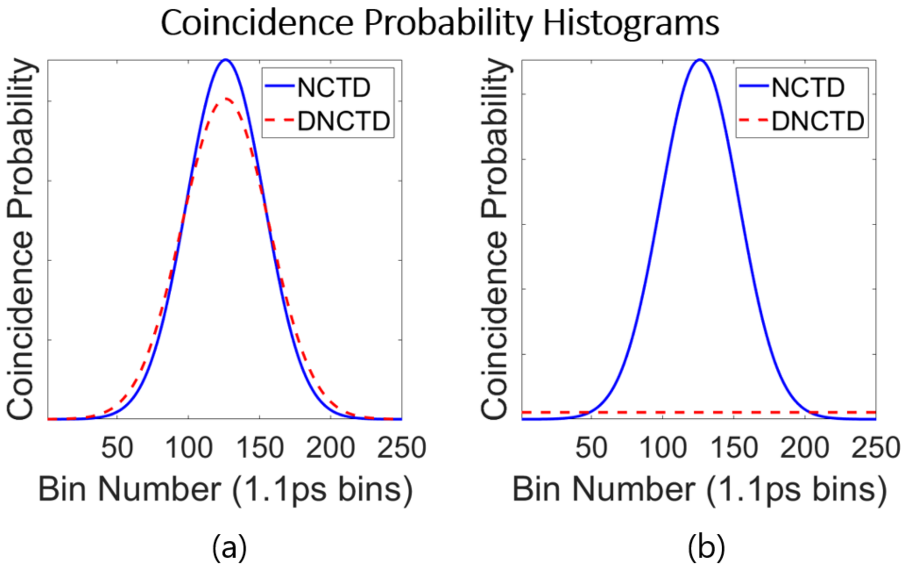

Supplementary Figure 3: Coincidence probability of true coincidence probability (a) and false coincidence probability (b) for a normalized noise power of $-13.9dB$.

The normalized noise power is calculated as the probability a noise photon is transmitted through the system divided by the probability a probe photon is transmitted.

$$\text{Normalized Noise Power} = \frac{\tau\tau_n^2 + (1-\tau)\tau_n^2}{\tau(1-\tau_n)^2} \tag{99}$$

This is equivalent to the experimental definition defined as the number of noise singles divided by the number of probe singles. In order to obtain the theoretical coincidence peak, for the nth time bin $\left(\Delta tn - \frac{\Delta t}{2}, \Delta tn + \frac{\Delta t}{2}\right)$ we integrate the joint probability function with respect to $t_p + t_r$ over all times and with respect to $t_p - t_r$ over the corresponding time bin. The corresponding marginal probability distribution yields the probability of obtaining a coincidence within each time bin. We can see from supplementary figure 3 that the disperersive fibers reduce the true coincidence histogram peak due to the non-maximal correlation between the probe and reference photons resulting from finite temporal duration of the pump. However, the noise coincidence probability is reduced substantially more resulting in a relative SNR improvement of $31.34dB - 13.837dB = 17.503dB$ using only the peak SNR values.

Repeating this calculation for many noise levels yields theoretical SNR curves for the relative improvement between the NCTD and DNCTD schemes. With the current normalization scheme the CTD and NCTD SNR are expected to be lines of slope -1 with no vertical offset. In order to obtain the improvement between the CTD and NCTD schemes we offset both the NCTD lines and DNCTD SNR curves from the CTD curve by the measured CTD to NCTD SNR improvement. The un-shifted lines are shown in the figure below for the same theoretical parameters as in supplementary figure 4.

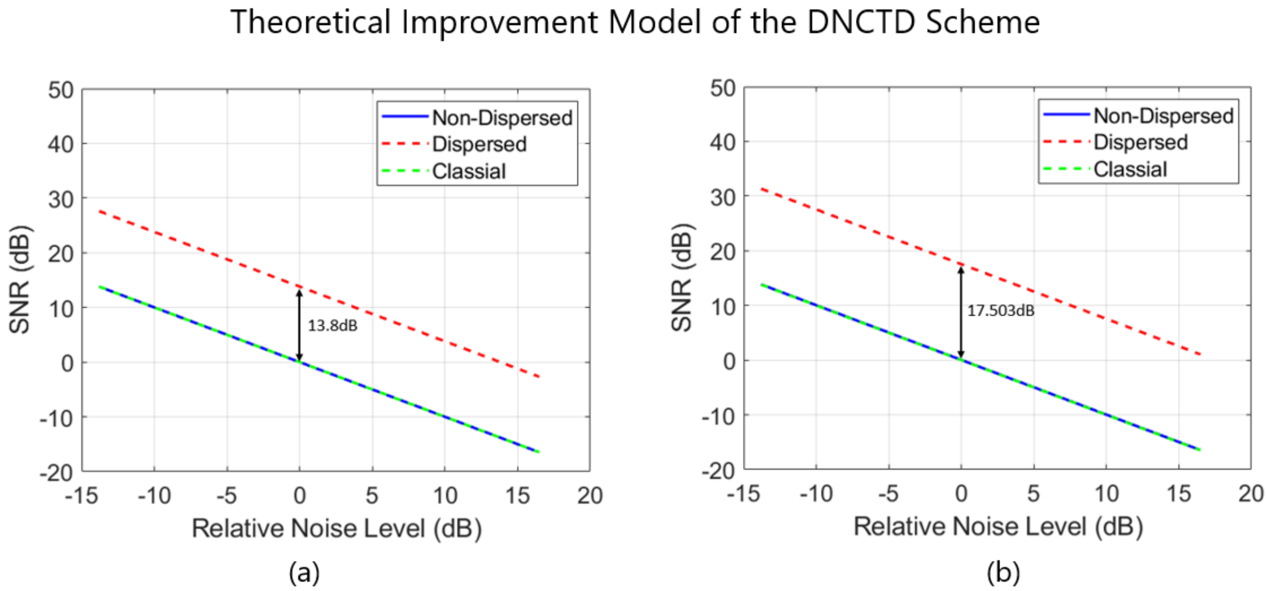

Supplementary Figure 4: Theoretical Modelling of Improvements by using the DNCTD Scheme (a) NCTD to DNCTD SNR improvement (Theoretical) calculated using maximum values of each coincidence peak. (b) NCTD to DNCTD SNR improvement (Theoretical) calculated with 200ps coincidence window.

Supplementary Note 14. Experimental Setup

The experimental setup used to demonstrate the viability of the DNCTD scheme over both the NCTD and CTD schemes is shown in 5. As the SPDC photons were created in a PPLN waveguide under Type 0 phase matching conditions the two photons have the same polarization and are in the same spatial mode. In order to deterministically separate the probe and reference photons we utilize the energy conservation relation that required $\omega_s + \omega_i = \omega_p$ where ω_s, ω_i, and ω_p are the frequencies of the signal, idler and pump respectively. By taking the reference photon from the pass port of a WDM with passband centered around $1570nm$ and sending the rejected light to a second WDM with passband centered around $1550nm$ from which the probe photons are taken, we can deterministically separate the signal and idler photons into two distinct paths.

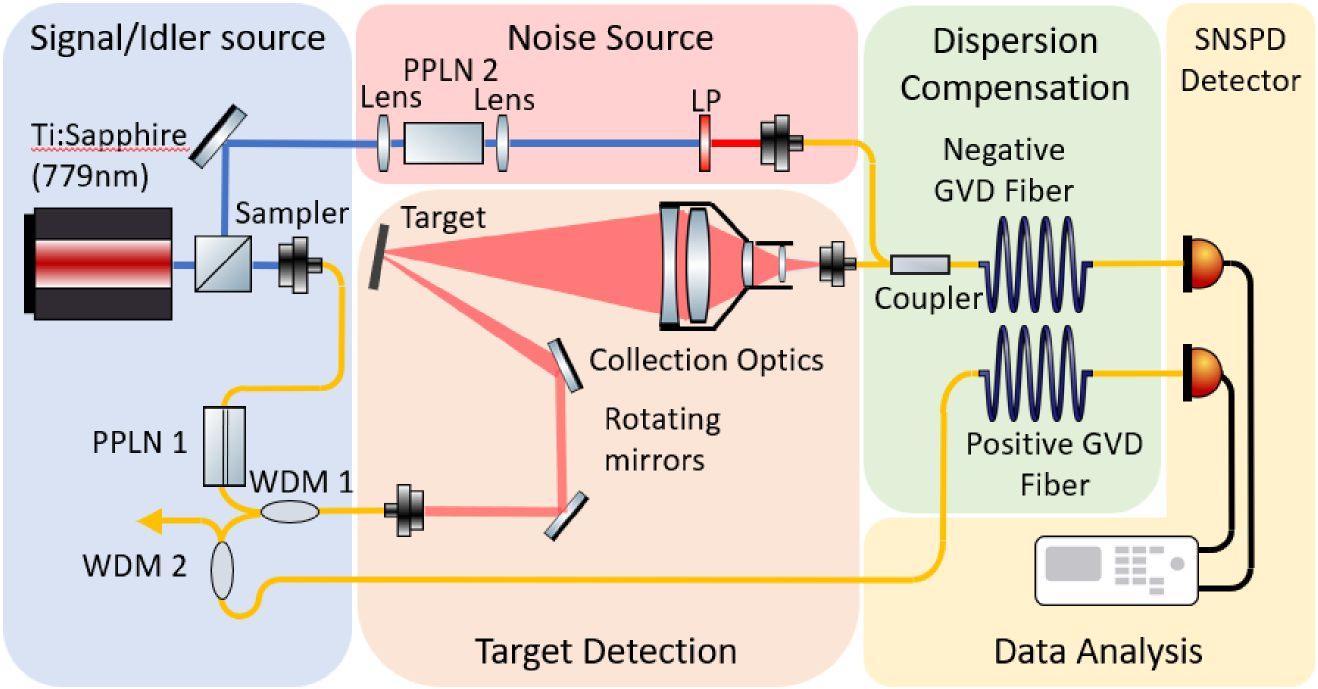

Supplementary Figure 5: Experimental Schematic for DNCTD scheme.

A Ti:sapphire oscillator (779nm,∼120fs, 80MHz) was split into two spatially separate paths; one to generate the temporally correlated probe-reference photons, and the other to generate the artificial noise source. The sampled arm is directed into a bulk PPLN crystal (PPLN 2) that serves to generate the noise photons that are nearly spectrally identical to the probe photons. A low pass filter (LPF) removes the remaining pump and the down-converted photons are collected into a fiber. The other arm is collected into fiber and directed to a fiber pig-tailed PPLN waveguide (PPLN 1) that serves as the probe-reference source. Two wavelength division multiplexers (WDM) serve to deterministically separate the probe and reference photons into two modes. The output from PPLN 1 is sent to the first wavelength division multiplexer (WDM) that passes the probe. The reflected arm is sent to another WDM (WDM2) that passes the reference beam. For the DNCTD scheme, the reference photon is immediately passed through a normal dispersion fiber (5km) on its way to the superconducting nanowire detector. This fiber is removed for the NCTD measurement. The probe photon is sent to a scanning setup which consists of rotating mirrors that can direct the beam onto different parts of a target. The three different targets consists of a tape 'U', 'O', and 'T', attached to the mirror put on an angle with the incident beam. The reflected light is then collected into fiber through an in-house designed telescope. The probe and noise are then combined at a 50/50 beam splitter and, for the DNCTD scheme, are passed through a dispersion shifted fiber (Negative GVD) and sent to another superconducting nanowire detector. Attenuation of the noise and probe is achieved by using a variable optical attenuator (VOA), before the dispersive fibers.

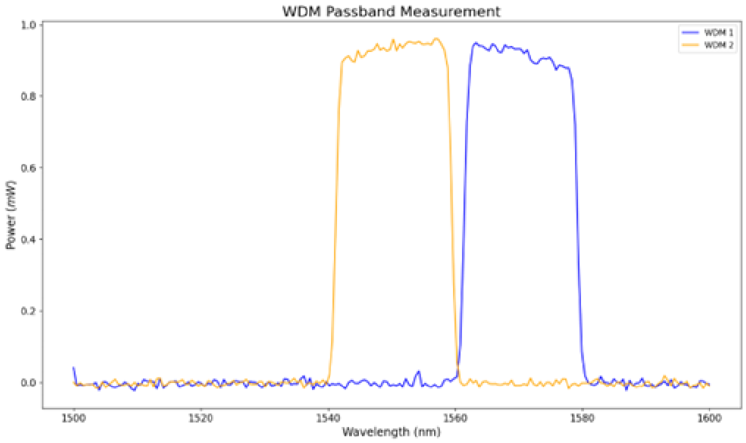

Supplementary Figure 6: Pass-band Characterization of WDMs used to separate probe and reference photons.

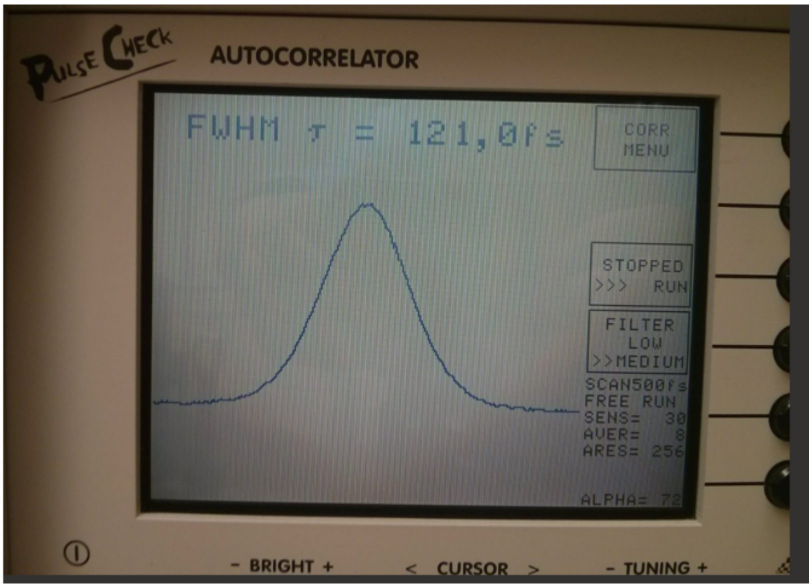

Supplementary Figure 7: Autocorrelation Measurement of pulses from Ti:Sapphire pump laser.

Supplementary Methods

SNR Measurement

To characterize the performance of the the DNCTD scheme compared with both the NCTD and CTD schemes we measured the signal to noise in four separate experiments. In the first experiment the noise power is varied using a VOA while in the probe power is held constant. In the second experiment, the VOA is moved from the noise arm to the probe arm and the probe power is varied while the noise power is held constant. In the third experiment, the

coincidence window is varied while all else is held constant. Finally, the SNR is measured for varying pump powers to show the effect of changing the pair rate on the performance difference between CTD and DNCTD. These two experiments are then repeated with the addition of the dispersive fibers. The method for determining the SNR from the measured quantities varies slightly for the different cases and is explained below.

Varying Noise Experiment

For the varying noise power experiment, we first collected coincidence data for 61 noise attenuation points each differing by 0.5dB from 0dB to 30dB as well as one additional point at 60dB that serves as a measure of the probe coincidences and singles. Coincidence data was taken for 10 seconds for each VOA attenuation level (Noise Power Level). For each point, the mean of the coincidence data was taken as the coincidence rate. This data includes all the probe and reference singles and coincidence data recorded by the time-tagger during the recording period. While we can estimate the probe singles and coincidences from this attenuation, we also performed the same experiment with the probe light blocked to exclusively record the noise singles and coincidences. The SNR for the CTD case is then calculated for each attenuation by

$$\text{Probe Singles} = \text{Total Singles (60dB Noise Attenuation)} \tag{100}$$

$$\text{Noise Singles} = \text{Total Singles - Probe Singles} \tag{101}$$

$$\text{SNR}_{\text{CTD}} = \frac{\text{Probe Singles}}{\text{Noise Singles}} \tag{102}$$

Note, probe singles are obtained from the 60dB noise attenuation measurement. For the NCTD and DNCTD schemes the SNR is calculated by

$$\text{True Coincidences} = \text{Coincidence (60dB Attenuation)} \tag{103}$$

$$\text{Noise Coincidences} = \text{Coincidences (Noise Measurement)} \tag{104}$$

$$\text{SNR}_{NCTD} = \text{SNR}_{DNCTD} = \frac{\text{True Coincidences}}{\text{Noise Coincidences}} \tag{105}$$

Where the true coincidences are calculated from the 60dB attenuation measurement and the noise coincidences are measured directly from the measurement where the probe light fiber was disconnected.

From the measured data, the pair rate was inferred from equation $SNR_{NCTD} = \frac{\nu\tau_p\tau_r}{N_b\nu\tau_r}$ and the singles rates Probe Singles $= \nu\tau_p$ and Ref Singles $= \nu\tau_r$ to be $\nu = 0.0035$ pair/pulse. The probe transmission was found to be 3.3% and the reference transmission 7.2%.

In order to calculate the improvement of the NCTD scheme over the CTD scheme a line was fit to the NCTD data using the variances to weight points. The slope was set to -1 resulting in a vertical offset of 28.788dB.

Varying Probe Experiment

In order to conduct the SNR measurement as we vary the probe channel loss, the VOA was moved from the noise path to the probe path (before the addition of noise). The loss in the probe arm was varied from 0dB to 15dB in increments of 0.25dB for a total of 60 measurements with an addition measurement made at 60dB attenuation to characterize the noise. Each measurement was conducted for 10s with a coincidence window of 200ps. The time, the noise light was blocked and another measurement was performed to characterize the probe singles and coincidences. In contrast to the varying noise measurements, it is now the probe singles and coincidences that become increasingly small. This means that, with high attenuation, the variance of the noise is relatively large compared with the probe and so to calculate the noise singles and coincidences by subtracting the 60dB attenuation measurement from the total. Thus the SNRs are calculated as

$$SNR_{CTD} = \frac{\text{Probe Singles - Probe Singles (60dB)}}{\text{Total Singles-(Probe Singles - Probe Singles(60dB))}} \tag{106}$$

Further, in order to calculate the probe singles we must subtract the background. This was not an issue in the previous case as the noise singles were calculated by subtracting measured quantities and so the background was implicitly subtracted and the probe singles at 60dB attenuation were much larger than the background making it insignificant.

$$SNR_{NCTD} = \frac{\text{Total Coincidences - Total Coincidences (60dB)}}{\text{Total Coincidences (60dB)}} \tag{107}$$

From the measured data, the pair rate was inferred in the same way as the previous section* and calculated to be $\nu = 0.00316$ pair/pulse. For the varying probe probe experiment, the maximum probe transmission was found to be 1.0% and the reference transmission 7.6%. The noise singles were measured to be around a constant 0.0404counts/pulse.

Varying Coincidence Window Experiment

Using a coincidence window that fully captures the coincidence peak does not lead to an optimal SNR for the DNCTD scheme. To observe the effect of changing coincidence window we performed coincidence measurements with coincidence windows from 10ps to 200ps in increments of 10ps. For each coincidence window width 100s worth of data was taken and averaged over to obtain the singles and coincidence results. The experiment was repeated with the probe disconnected to characterise the noise. The SNRs were calculated according to

$$SNR_{NCTD/DNCTD} = \frac{\text{Total Coincidences - Noise Coincidences}}{\text{Noise Coincidences}}.$$

From the largest coincidence window (200ps) which captures essentially all of the coincidence peak. For this measurement the pair rate was set on the order of the background rate and so the pair rate is difficult to estimate accurately as the probe and reference singles counts cannot be well distinguished from background. Nevertheless the coincidence counts are still easily countable as the coincidence background is essentially zero and this measurement does not compare with the CTD scheme and the knowledge of the singles does not change the result. For this experiment, the noise singles were 0.019 counts/pulse.

Varying Pump Power Experiment

In order to achieve the largest possible SNRs difference between the CTD and DNCTD schemes, we reduce the SPDC pair generation rate as low as possible while using a 10ps coincidence window. The SNR was measured for 8 different pump power levels (adjusted with a continuously variable attenuation wheel). Each measurement consisted of 500s worth of data for a 10ps coincidence window with no noise attenuation, 250s worth of data for a 10ps coincidence window with 60dB of noise attenuation, 250s worth of data with a 200ps coincidence window wtih 60dB of noise attenuation. The first two measurements are used to characterise the total, probe, and noise coincidence and singles data while the last measurement is used to infer the pair rate. The SNR is calculated as

$$\text{Probe Singles} = \text{Singles (60 db Noise Attenuation) - Background Singles} \tag{108}$$

$$\text{Noise Singles} = \text{Singles (0 dB Noise Attenuation) - Probe Singles} \tag{109}$$

$$\text{True Coincidences} = \text{Coincidences (60 db Noise Attenuation)} \tag{110}$$

$$\text{Noise Coincidences} = \text{Coincidences (0 db Noise Attenuation)} \tag{111}$$

$$\text{SNR}_{\text{CTD}} = \frac{\text{Probe Singles}}{\text{Noise Singles}} \tag{112}$$

$$SNR_{NCTD,DNCTD} = \frac{\text{True Coincidences}}{\text{Noise Coincidences}} \tag{113}$$

In order to compare each of the data points we need a metric for the pump power used. The pair rate suffices well however to calculate it we need to know the full number of coincidences not only the section* obtained when using a 10ps window. Using the 200ps coincidence window measurement, the pair rate is calculated according to equations

$$\text{Reference Singles} = \text{Reference Singles - Background Reference Singles} \tag{114}$$

$$\text{Probe Singles} = \text{Probe Singles - Background Probe Singles} \tag{115}$$

$$\text{True Coincidences} = \text{Coincidences (60db Noise Attenuation, 200ps Coincidence Window)} \tag{116}$$

$$\text{Pair Rate} = \frac{\text{Probe Singles} \times \text{Reference Singles}}{\text{Coincidences}} \tag{117}$$

Collection Optics and Telescope Design

In order to allow for 3D scanning of the environment using the DNCTD scheme, a uniquely designed wide angle collection system was necessary. This is necessitated by maintaining a high advantage over the CTD scheme in a large regions of interest. In order to achieve the advantage in the quantum LiDAR setup, one must use a single mode fiber to limit the photons to a single mode to allow the use of the super conducting nano-wire detector in the DNCTD scheme. This however limits the detection setup to a single pixel, allowing the setup to only be able to detect whether a target is present or not.

Adding scanning capabilities to the setup will allow for the ability to do low photon imaging of the target plane. Although this has been done with transmission of light through a target, imaging with signal idler photon pairs by reflection is a new and unique technology. This would also allow for the imaging and detection of targets in high noise environments without the need for powerful light sources. This technology is extremely attractive for technologies in self-driving vehicles, covert military detection, satellite communication, and biological imaging, where a large photon flux can damage the sample.

In order to achieve the goal of creating the multi-pixel setup, there are two main features that must be added: Wide angle collection; and a scanning mechanism. Wide-angle collection refers to the necessity to create a system that will be able to couple light into the single mode fiber, while maintain a high coupling efficiency. Scanning mechanism is simply the setup that will allow the single pixel detection to raster scan across an area of interest and image the intended target. The following section*s will describe in more detail the approach towards these solutions.

Collection

It is not possible to maximize the coupling efficiency for multiple angles, as the numerical aperture of the single mode fiber is fixed. We can however reduce the loss due to mode mismatch. This is a difficult task due to the set numerical aperture of the single mode fiber. Multiple designs were created using ZEMAX software to simulate the coupling of light into a single mode fiber using a telescope. The inspiration of the design was to use a negative meniscus lens (commonly found in wide angle lens and fisheye lens) due to their prevalent use in wide angle systems. A Catadioptric (Cassegrain) scope would not be viable in this situation as they require the incoming light to come from a distant target (so that the light entering the telescope is approximately collimated. Using this design, models of various negative meniscus setups were created in ZEMAX software and tested in order to compare their performance. Supplementary figure 8 illustrates an example of the setup used in ZEMAX to simulate the lab environment.

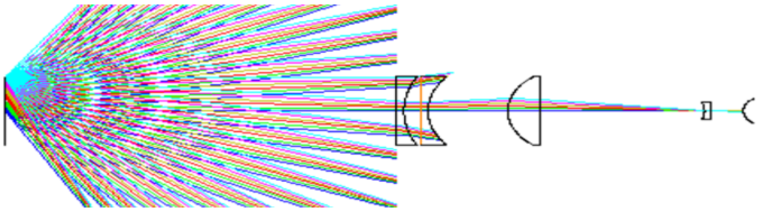

Supplementary Figure 8: Experimental setup in Zemax to test collection of a radiating point source at different off-angles with the telescope

Supplementary figure 8 graphs the theoretical coupling efficiencies from point sources one meter from the telescope at various displacements from the on-axis. The figure shows the improvement over the original telescope used, as well as the ability of the meniscus lens to couple from wide angles.

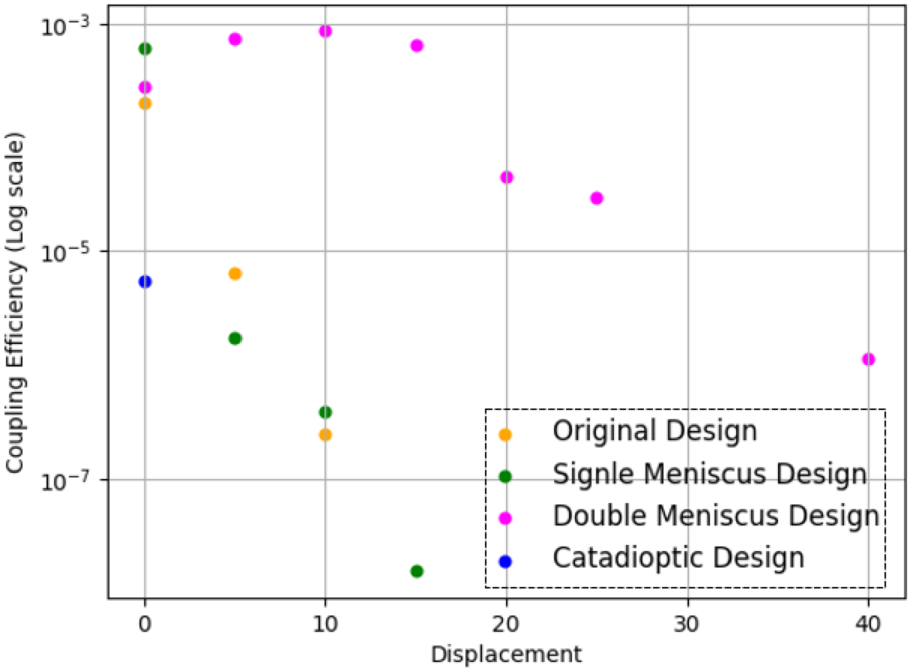

Supplementary Figure 9: Results of the testing of telescope designs within Zemax for the original,single meniscus, double meniscus, and catadioptric lens setups

The original design was the telescope used in the previous experiment and consisted of plano-convex lens. The exact lens used in the original setup and the double meniscus setup is listed in the table below

Telescope	Lens in 2" section*	lens in 1/2" section*
Original	f = 200.0 mm plano-convex lens<br>f = 60.0 mm plano-convex lens	f = -25 mm plano-concave lens<br>f = 15.0 mm plano-convex lens
Double Meniscus lens	f = -100.00 mm meniscus concave lens<br>f = -75.00 mm meniscus concave lens<br>f = 60.00 mm plano convex lens	f = -30 mm plano-concave lens<br>f = 15.0 mm plano-convex lens

Supplementary Table 1: Comparison of telescope designs using different lenses.

Scanning

Raster scanning requires the ability to steer the beam across the x-y plane in order to sequentially scan across the entire target. In order to do so, two rotating galvanometer mirrors are used. These were chosen due to their low loss and ease of implementation onto the setup. By changing the voltage sent to the galvanometer controller, one can change the angle of the galvanometer mirror. One mirror is used to scan the x-direction while the other mirror scans the y-direction. The model of galvanometer used was the Thorlabs Inc. GVS001.

Scanning test

A large scale test scan was done to show the imaging capabilities of the setup. The target chosen for the test experiment is an aluminum target. On the aluminum target, a black "UofT" was drawn to be imaged. Although it does not have the same diffusivity of a more realistic target, aluminum is a better representation than a mirror. The source for the experiment was a 1550nm LED, and the detector used was a Germanium detector to measure the power coupled from the telescope. The results can be shown in supplementary figure 10, which depict the ability to resolve the "UofT" in a 12 x 56 pixel image, as well as the ability to fully resolve each letter.

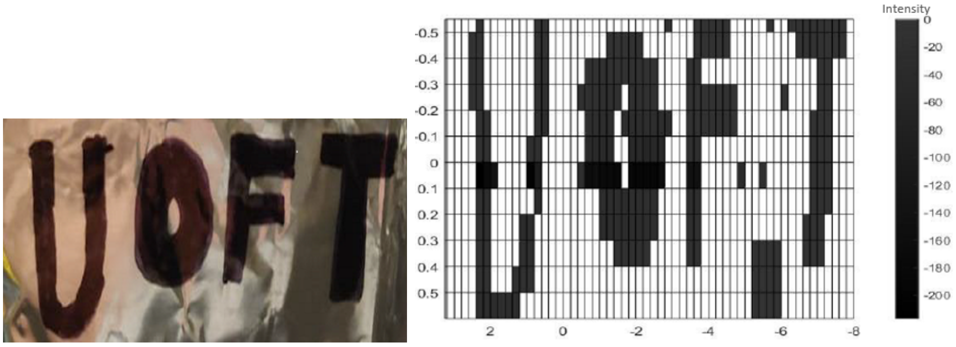

(a) (b)

Supplementary Figure 10: Scan done of "UofT" on a piece of aluminum foil using a basic single pixel LiDAR setup. (a) Original Image. (b)Scanned image

Integration of DNCTD scheme with imaging setup

The integration with the DNCTD scheme was done by connecting the probe photon path from the first PPLN in supplementary figure 5 to a collimating lens which directs the probe photons onto the rotating galvonometer mirrors. The mirrors scan the probe onto the target, and the reflected light is coupled from the telescope into a single mode fiber. The single mode fiber is connected to the negative GVD fiber and finally the superconducting nanowire detector.

Depth is measured by using the time delay from the coincidence bins. As the target moves further away, the time traveled by the photons increase and consequently the time delay increases. Knowing the speed of photons, the relative change in delay can be used to measure the depths of the image, with the resolution being the bin width of the coincidence bins (which in this experiment were 3ps).

In order to do 3D imaging, three letter targets (”U”, ”O” ,and ”T”) were placed in the field of view of the telescope. The targets are simply unreflective tape letters placed onto mirrors. The rotating galvanometer mirrors raster scan the mirrors in order to image the letters on them.

Supplementary References

[1] Miguel A Alonso. Wigner functions in optics: describing beams as ray bundles and pulses as particle ensembles. *Advances in Optics and Photonics*, 3(4):272–365, 2011.

[2] Keisuke Goda and Bahram Jalali. Dispersive fourier transformation for fast continuous single-shot measurements. *Nature Photonics*, 7(2):102–112, 2013.

[3] Sébastien Coëtmellec, Marc Brunel, Denis Lebrun, and Jean-Bernard Lecourt. Group velocity dispersion with fractional-order fourier formalism. *Journal of Optics A: Pure and Applied Optics*, 5(1):26, 2002.

[4] Marc Brunel, Sébastien Coetmellec, Mickael Lelek, and Frédéric Louradour. Fractional-order fourier analysis for ultrashort pulse characterization. *JOSA A*, 24(6):1641–1646, 2007.

[5] Yingwen Zhang, Duncan England, Andrei Nomerotski, Peter Svihra, Steven Ferrante, Paul Hockett, and Benjamin Sussman. Multidimensional quantum-enhanced target detection via spectrotemporal-correlation measurements. *Physical Review A*, 101(5):053808, 2020.

[6] Lynden K Shalm, Deny R Hamel, Zhizhong Yan, Christoph Simon, Kevin J Resch, and Thomas Jennewein. Three-photon energy–time entanglement. *Nature Physics*, 9(1):19–22, 2013.

[7] Malte Avenhaus, Andreas Eckstein, Peter J Mosley, and Christine Silberhorn. Fiber-assisted single-photon spectrograph. *Optics letters*, 34(18):2873–2875, 2009.

[8] Jacob Mower, Zheshen Zhang, Pierre Desjardins, Catherine Lee, Jeffrey H Shapiro, and Dirk Englund. High-dimensional quantum key distribution using dispersive optics. *Physical Review A*, 87(6):062322, 2013.

[9] JD Franson. Nonlocal cancellation of dispersion. *Physical Review A*, 45(5):3126, 1992.

[10] Haldun M Ozaktas, M Alper Kutay, and David Mendlovic. Introduction to the fractional fourier transform and its applications. In *Advances in imaging and electron physics*, volume 106, pages 239–291. Elsevier, 1999.

[11] Vahid Ansari, John M Donohue, Benjamin Brecht, and Christine Silberhorn. Tailoring nonlinear processes for quantum optics with pulsed temporal-mode encodings. *Optica*, 5(5):534–550, 2018.

[12] Wojciech Wasilewski, Alexander I Lvovsky, Konrad Banaszek, and Czesław Radzewicz. Pulsed squeezed light: Simultaneous squeezing of multiple modes. *Physical Review A*, 73(6):063819, 2006.
